# Milk allergy and bottles over the back fence: two single patient trials

**DOI:** 10.1186/1757-1626-1-77

**Published:** 2008-08-08

**Authors:** Bruce Arroll, Harry Pert, Gordon Guyatt

**Affiliations:** 1 Department of General Practice and Primary Health Care, Faculty of Medical & Health Sciences, University of Auckland, New Zealand; 2 Ranolf Medical Centre, Rotorua, New Zealand; 3 Department of Clinical Epidemiology and Biostatistics, McMaster University, Hamilton, Ontario Canada

## Abstract

**Objective:**

To determine if allergy to cows milk was responsible for symptoms in two children.

**Design:**

Single patient trial.

**Setting:**

General Practice in New Zealand.

**Participants:**

Two children aged about 6 months

**Intervention:**

Alternating bottles of soya-based milk and cow's milk provided by neighbours over their back fence.

**Main outcome measures:**

Presence of diarrhea, irritability, rash and wheeze.

**Results:**

After 4 cycles of soya-based milk and cows milk one child proved to have a milk allergy and one did not.

**Conclusion:**

A systematic approach enabled conclusive diagnoses in both children.

## Introduction

Parents of young children whose behaviour is not conforming to expected patterns are often concerned about milk allergy. Children who acquire milk allergy typically do so in the first one to two years of life and commonly lose the allergy after a few months [1]. Two children, one aged about 6 months presented to their general practitioner with a history of diarrhea, skin rash, irritability and wheeze. The parents, reinforced by their peer group's opinions, were considering milk allergy as a possible cause of the problems. Both sets of parents remained uncertain, and proved open to a single patient randomized trial to resolve the issue [2,3]. This study was conducted more than twenty years ago but came to light when the GP (HP) was in a research course discussing single patient trials. Hence there is no record of quantitative data but the authors thought that this was such a good example of using a systematic approach that we should share it with our colleagues. The particular example has always been warmly received when presented to groups of general practitioners and other health professionals.

## Case presentation

The two children were aged about 6 months. They had no significant medical history.

They both presented with diarrhoea, red rash on their skin, irritability and wheeze. Both sets of parents thought that milk allergy could be a cause for their symptoms. No specific diagnostic tests were conducted and no other interventions were tried.

The children underwent four pairs of treatment periods; within each pair, the children received soy-based milk during one period, and cow's milk during the other. Within pairs a coin flip determined the order of the two preparations [3]. As symptoms appeared to come and go within 48 hours of consuming cows milk cycles of 4 days it was considered sufficient to use the onset of symptoms to assess the clinical effects.

Both families' neighbours, who agreed to supply bottles of milk containing either soya-based products or cow's milk based products, received the respective allocation schedules. To ensure blinding of parents, the neighbours prepared the milk in standard bottles and passed them over the back fence. The families knew neither what type of milk each bottle contained, nor how long each treatment period would be. The children did not seem to notice the difference in the taste of the milk and the rest of their diet remained constant. The families kept diaries of the concerning symptoms i.e. diarrhea, skin rash, irritability and wheeze. The neighbours kept a diary of the contents of each milk bottle. To evaluate the results, the general practitioner (GP) met with the patients and compared their diary findings with that of the milk contents from the neighbours' records. There was no concern about anaphylaxis to the soya-based milk as this is an uncommon event. The parents were advised not to change any other aspects of their children's care.

The two families brought the results of the study along with the neighbours schedule to their GP (HP). Results from one of the children demonstrated no relationship with the type of milk consumed. The parents were very relieved and continued their child on cow's milk products. Results from the other child showed an evident relationship between cow's milk and the concerning symptoms. The symptoms came on within 24 hours of starting the cows milk. The family proceeded to remove all cow's milk containing food from the child's diet. Both sets of parents were relieved by the outcome. It meant that the family with the milk allergy could continue with the difficult task of avoiding milk products knowing that it was worthwhile. The family with the child without a milk allergy were also relieved that they did not have to continue with the milk avoidance.

## Discussion

A Medline search of "community involvement" and "milk allergy" revealed no relevant papers, suggesting this may be the first published case of recruitment of a neighbour to assist with diagnosis of milk allergy. The single patient trial methodology lends itself to clarifying an issue such as possible milk allergy. The issue is important for the family, the onset and termination of action is rapid, and symptoms easily measured.

The novel use of a neighbour enabled parents to remain blind to the type of milk their child received [2]. Blinding of parents to the duration of each period provided a further safeguard against bias. It does not really matter if the parents could be unblinded as it is not in their interest to "cheat." They did not have to embark on the study and hence had no incentive to break the rules. The children may have been able to detect the difference in taste but they would not have been able to exhibit the symptoms and signs in accordance with the different forms of milk. The clarity of the results – in one case showing no relationship between formulation and symptoms, in the other a clear relationship – obviated the need for formal statistical analysis.

These experiments demonstrate the value of a systematic approach to suspected milk allergy in children. The studies also demonstrate how one can tailor single subject experiments (N of 1 trials) to individual patients. Not only did the trials address the specific issues of the parents, the design took advantage of the families' social supports. In doing so, the study took advantage, and perhaps further fostered, the community's social solidarity.

## Competing interests

The authors declare that they have no competing interests.

## Authors' contributions

HP was the GP in the two cases. BA wrote the first draft and GG recognised the importance of this study and encourage HP to publish. All three authors commented on the first and subsequent drafts.

## Consent

This work occurred more than 20 years ago and the patients are no longer known or contactable.
